# An optimal dose‐fractionation for stereotactic body radiotherapy in peripherally, centrally and ultracentrally located early‐stage non‐small lung cancer

**DOI:** 10.1111/1759-7714.15071

**Published:** 2023-09-10

**Authors:** Izabela Zarębska, Maciej Harat

**Affiliations:** ^1^ Department of Neurooncology and Radiosurgery Franciszek Lukaszczyk Oncology Center Bydgoszcz Poland; ^2^ Department of Radiotherapy Franciszek Lukaszczyk Oncology Center Bydgoszcz Poland; ^3^ Center of Medical Sciences University of Science and Technology Bydgoszcz Poland

**Keywords:** early‐stage non‐small lung cancer (NSCLC), SABR, SBRT, stereotactic ablative radiotherapy, stereotactic body radiation therapy

## Abstract

Stereotactic body radiotherapy (SBRT), also known as stereotactic ablative radiotherapy (SABR), is commonly used in inoperable patients with early‐stage non‐small lung cancer (NSCLC). This treatment has good outcomes and low toxicity in peripherally located tumors. However, in lesions which are located close to structures such as the bronchial tree or mediastinum the risk of severe toxicity increases. This review summarizes the evidence of dose‐fractionation in SBRT of NSCLC patients in various locations.

## Graphical abstract

1. In peripheral tumors shorter interval between fractions does not increase toxicity.
2. Treatment on consecutive days is recommended.
3. In peripheral tumors up to 5 cm radiosurgery is recommended. In tumors abutting chest wall – 5 × 11 Gy
4. In central tumors >1 cm from main bronchi 5 × 10 Gy, in ultracentral tumors 8 × 7.5 Gy. In gross endobronchial infiltration the schedule of 8 × 7 Gy should be avoided. In coexistence of risk factors for inducing hemoptysis such patients should be treated with extreme caution.
5. In ultracentral tumors short schedules of <3 fractions should be avoided.
6. Re‐SBRT is often used but it should be avoided in ILD and only >6 months after prior treatment.

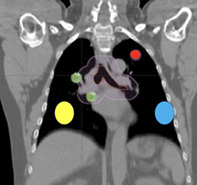

Stereotactic radiotherapy in early‐stage non‐small lung cancer has excellent local control and low toxicity in peripheral tumors. In central and ultracentral location this treatment may cause severe toxicity. Optimal dose‐fractionation adopted to organs at risk increases the safety of radiotherapy in these localisations.

## INTRODUCTION

Lung neoplasms are the leading cause of cancer mortality worldwide. According to GLOBOCAN 2020 statistics, lung cancer is the main cause of death in both men and women (18% of total cancer deaths).^1^, ^2^ The most common lung cancer type is non‐small cell lung cancer (NSCLC) representing 85% of cases. Its main pathological subtypes are adenocarcinoma, squamous cell carcinoma and large cell carcinoma. Small cell lung cancer (SCLC) is a less common neoplasm, associated with 15% of lung cancer cases.^3^

Surgical resection is the standard of care for patients with early‐stage NSCLC (stage I and II, T1‐2N0M0).^4^ Among the large group of inoperable patients or those who refuse surgery, stereotactic body radiation therapy (SBRT), also known as stereotactic ablative radiotherapy (SABR), has shown excellent outcomes with low toxicity. SBRT is a noninvasive treatment that precisely delivers high radiation doses to small targets sparing healthy tissues usually in <5 fractions.^5^, ^6^, ^7^ Long‐term survival after SABR is comparable to video‐assisted thoracoscopic surgical (VATS) lobectomy with mediastinal lymph node dissection (VATS L‐MLND) in patients with operable stage IA NSCLC.^8^ The main obstacle of lung SBRT is connected with tumor location. SABR has been commonly used for peripherally located tumors—lesions >2 cm in all directions from the central airways—trachea, carina and main bronchus up to the division of the second order bronchi.^9^ Based on existing evidence (level II), an increased biological effective dose (BED) (e.g., 3 × 20 Gy) resulted in a higher rate of grade 3 toxicity. High rates of local control, grade 3 toxicity in >10% patients and incidental higher toxicities (deaths included) suggest optimal dose‐fractionation should be selected for specific SBRT cases.

Centrally located cancer is defined as a lesion located <2 cm from the proximal bronchial tree. In an ultracentral location gross tumor volume (GTV) invades the proximal bronchial tree or mediastinal structures. These central and ultracentral locations have previously been defined as the “no‐fly zone” based on excessive toxicity in an early phase II trial.^10^, ^11^ However, optimized dose‐fractionation adapted to organs at risk tolerance increase safety of SBRT in central locations.^12^

In ultracentral location patients, the role of SBRT has recently been presented in ISRS guidelines and reported to be safe with appropriate patient selection, consideration of concomitant therapies, and radiotherapy plan design.^13^ [Correction added on 26 September 2023, after first online publication: Yan et al., 2023 has been added in the References list. The reference citations were renumbered accordingly.]

Many fractionation schedules have been reported so far, but the optimal treatment schedules are still unclear. There is still nuance attention needed: radiotherapy on consecutive days, single fraction treatment or reirradiation.

In this review, we summarize recent evidence of SBRT dose‐fractionation schemes and its toxicity in peripheral, central and ultracentral early‐stage NSCLC in relation to these factors.

## METHODS

The authors performed a Pubmed search of published journal articles written in English. We used keywords “SBRT”, “SABR”, “peripheral early‐stage non‐small cell lung carcinoma” or “ultracentral/central early‐stage non‐small cell lung carcinoma”. We included retrospective studies, prospective studies and systematic reviews.

### SBRT in peripheral locations—where are we now?

The ESTRO‐ACROP consensus from 2017 summarizes recommendations of SBRT in peripheral early NSCLC. The consensus recommends 3 × 15 Gy, 113 Gy biological effective dose (BED) and 4 × 12 Gy for lesions with broad chest wall contact (BED10 106 Gy). The consensus does not define strict recommendations regarding intervals between fractions. These schedules have been evaluated in prospective phase II trials and achieved local control of 90%. The treatment is usually very well tolerated, with most toxicities being grade 1–2. The consensus also recommended 3 × 18 Gy for patients with favorable OS expectations and without severe comorbidities.^14^, ^15^, ^16^

A recent retrospective study has evaluated SBRT schemes with the emphasis on dose interval. Various fractionation schemes have been used: 5 × 11 Gy on alternate days and 8 × 7.5 Gy on consecutive days in tumors located <1 cm from the chest wall or 3 × 18 Gy on alternate days in tumors >1 cm from the chest wall. In patients with toxicity concerns or unachievable dose constraints the authors prescribed 10 × 5 Gy (BED 75 Gy) on consecutive days. The median OS for all patients was 42.3 months, with 3‐ and 5‐year OS rates of 52.8% and 37.3%, respectively. There were no statistical differences between tumor location and survival. However, the schedule of 10 × 5 Gy as well as poor performance status were connected with significantly poorer survival. The treatment was usually well tolerated with 1% rate of 3–4 toxicity.^17^

A randomized pilot study compared acute toxicity of peripheral tumors treated with SBRT delivered in 4 or 11 days. The prescribed treatment was 12–13 Gy in four fractions. Patients treated over four consecutive days experienced more grade 2 or higher acute toxicity; however, the results were statistically insignificant. Dyspnea, fatigue and cough were worse in the 11 day group. More patients treated on four consecutive days experienced an increase in the dyspnea at 1 and 4 months after treatment, compared to those treated over 11 days.^18^

### Single fraction lung SBRT (SF‐SBRT)

A retrospective study showed that SF‐SBRT of 1 × 30 Gy was similar to 3 × 16–20 Gy in terms of OS, LC and toxicity. In this study, more patients with Karnofsky performance status (KPS) score <80 were in the single‐fraction group. Characteristics such as patients age, smoking status, tumor stage, tumor size and histological type were comparable between groups. One‐ and 2‐year OS rates were 86.1% and 63.2%, respectively for the single fraction cohort, and 80.8% and 61.6%, respectively for the three fraction cohort. One‐ and 2‐year LC rates were 95.1% and 87.8%, respectively for the single fraction cohort, and 92.7% and 86.2%, respectively for the three fraction cohort. There was only one grade 3 pulmonary embolism in the three fraction cohort.^19^

The scheme of 1 × 30 Gy versus 3 × 20 Gy with at least 40 h intervals between each fraction has been studied in a randomized phase 2 trial. Median follow‐up was 53.8 months. Grade 3+ thoracic toxicities occurred in 17% patients (30 Gy) and in 15% patients (3 × 20 Gy). Grade 4 and 5 toxicities was not observed. OS at 2 years was 73% for arm 1 and 62% for arm 2. PFS at 2 years was 65% for arm 1 and 50% for arm 2. There were no statistical differences in LC between arms. LC at 2 years was 94.9% in arm 1 and 97.1% in arm 2.^20^

NRG Oncology RTOG 0915 (NCCTG N0927), a randomized phase II study, tested 1 × 34 Gy (arm 1) or 4 × 12 Gy on consecutive days (arm 2). The aim of this study was to evaluate which schedule causes less cases of grade 3+ adverse effects at 1 year. Arms were balanced in terms of treatment and patients characteristics. Both schedules were safe with toxicities usually grade <2. Endpoint adverse effects experienced changes in pulmonary function and pneumonitis were present in 10.3% patients from arm 1 and 13.3% from arm 2 including mostly changes in pulmonary function. There was no significant difference in terms of survival. Two‐year OS was 61.3% (arm 1) and 77.7% (arm 2). Local control at 1 year was 97.0% (arm 1) and 92.7% (arm 2).^21^ Long‐term results of this study revealed that in arm 1 – 2.6% patients and in arm 2 –11.1% patients experienced treatment‐related grade 3 and higher toxicity. Four‐year OS was comparable but the biggest difference between both arms was in 5‐year OS (29.6% [arm 1] and 41.1% [arm 2]). The difference may have resulted from a small number of patients in follow‐up. Authors suggested SF‐SBRT as appropriate treatment in early NSCLC.^22^

In a long‐term prospective study, SF‐SBRT doses of 30 Gy and 34 Gy were well tolerated with good patient outcomes. Median OS was 44.1 months in 30 Gy and 42.8 in 34 Gy patients. Overall toxicities were observed in 27% of patients, with no grade 4/5 toxicity. There were no statistical differences in toxicities between dose cohorts. The most common symptoms included grade 1–2 pneumonitis in 7% of patients with no difference depending on dose schedule. Chest wall toxicity (grade 1–3) was observed in 13.5% of patients with no statistical difference between schedules. Grade 3 effusion was observed in 0.4% of the 1 × 30 Gy group. No other grade 3 and higher toxicities were present.^23^

In the abovementioned studies in most cases the technique used was 4D‐CT. Non‐4D‐CT simulation was utilized when tumor motion was <5 mm. In that case the craniocaudal margin of 1 cm was added.

A phase II trial of individualized stereotactic ablative radiotherapy for lung tumors showed that small peripheral tumors up to 10 cc can be treated with 1 × 25 Gy (BED <100 Gy). However, the final results of the trial have not yet been published.^24^

The results support single dose treatment in older patients with poor performance status, unfit for surgery or those who refuse surgical resection with tumors <5 cm. Stereotactic radiosurgery is ideal for patients with poor reproducibility of treatment position or poor access to transportation. SF‐SBRT offers short time of treatment, high quality of life with favorable outcomes and low rates of toxicity. A systematic review from 2022 supports SF‐SBRT in the treatment of early NSCLC.^25^

### Summary of evidence for SBRT in peripheral tumors

Few studies evaluating schedules other than those presented in the ESTRO consensus from 2017 have been published in the last years. Existing evidence suggests that treatment on consecutive days has similar efficacy and toxicity to well‐known schedules. Moreover, single dose treatment based especially on 1 × 30 Gy schedule also does not increase toxicity in comparison to hypofractionation schedules and is not less effective than 1 × 34 Gy scheme. The results of 1 × 25 Gy are encouraging. However, further studies are needed to confirm its efficacy and safety. In clinical practice more conservative schedules should be used.

### Current status of SBRT in central and ultracentral lung tumors

A systematic review from 2019 summarized the data of 250 patients with ultracentral lung tumors. Dose‐fractionation schedules included schedules of 3–12 fractions up to 30–60 Gy. BED10 ranged from 48 to 180 Gy. Median follow‐up was 20 months. Median treatment‐related toxicity of grade 3+ was 10%. It included pneumonitis, esophagitis, atelectasis, bronchitis, bronchial stricture, dyspnea, and hemoptysis. Median treatment‐related mortality was 5% caused by pulmonary hemorrhage, pneumonia, respiratory failure, bronchial fistula, bronchial stricture, bronchial obstruction and heart failure. High‐risk indicators of SABR‐related mortality were associated with gross endobronchial disease, maximum dose to the proximal bronchial tree greater than or equal to 180 Gy (which corresponds to 45 Gy in 5 fractions or 55 in 8 fractions), peri‐SABR bevacizumab use, and antiplatelet/anticoagulant use. Median 1‐year local control rate was 96% and median 2‐year local control rate was 92%. Studies with lower rates of 1‐year local control (63% and 70%) and reduced toxicity included schedules of 5 × 8 Gy, BED = 72 Gy.^26^

According to the ASTRO consensus, schemes of three fractions should be avoided in a central location. Preferred treatment with an acceptable safety profile and low incidence of toxicity includes 4–5 fractions. In high risk (ultracentral) cases, where dose constraints could not be met, hypofractionated schedules of 6–15 fractions should be considered.^27^

Another systematic review and meta‐analysis showed that patients with ultracentral tumors receiving SBRT in 4–12 fractions up to 40–60 Gy (BED10 85.5–132) had 2‐year LC 96.7%, 2‐year OS 57.7% and grade 3+ complication rate 23.2%. Grade 5 toxicity incidence among the studies was 0%–22%, with hemorrhage as the most common fatal complication (68,2%). The risk factors for fatal hemoptysis included anticoagulant use, excessive maximum irradiation dose, endobronchial involvement, squamous histology, and bevacizumab use.^28^

The prospective phase I/II trial by Roach et al. revealed that 11 Gy in five fractions had 2‐year LC 85% and 2‐year OS of 43%. The rate of grade 3+ acute toxicity was 6%, with no cases of grade 5 events. It included pulmonary events, malnutrition and dehydration. Late cardiac and pulmonary toxicity was present in 27% (grade 3), 12% (grade 4) and 4% (grade 5) patients. The toxicities included myocardial infarction and pneumonia. Grade 5 death was caused by bronchial stenosis and fatal hemoptysis.^29^

The HILUS TRIAL revealed that patients with ultracentral lung tumors treated with 56 Gy in eight fractions (7 Gy per fraction, prescribed to 67% isodose) delivered in 2 weeks experienced substantial complications. Cohort A included lesions located 1 cm and less from main bronchi and trachea, and cohort B included the rest of the cases. Grade 3 to 5 toxicity was present in 34% patients and 15% experienced grade 5 toxicity (10 patients died from treatment‐related bronchopulmonary hemorrhage, pneumonitis and fistula). As expected, patients from cohort A were at higher risk of adverse events. The authors recommend avoiding this treatment in patients with lesions within 1 cm from the main bronchi and trachea. Tumors from patients in cohort B might be treated with this dose‐fractionation schedule. However, treatment must be based on individual assessment and maximum dose to the main bronchi and trachea in the order of 70 to 80 Gy (equivalent dose in 2 Gy fractions).^30^

Patients unfit for surgery or SBRT may benefit from 60 Gy in 15 fractions over 3 weeks. Compared to 60 Gy in 20 fractions this schedule was connected with a favorable safety profile—with no grade 4–5 pneumonitis and no grade 2–5 esophagitis.^31^

SBRT in seven fractions of 10 Gy in central tumors had similar outcomes in terms of OS, PFS, local recurrence, regional recurrence, or distant metastasis compared with 50 Gy in four fractions.^32^

The CALBG trial prescribed 70 Gy in 17–29 fractions with 31% complete response and 46% partial response. The median OS of the treatment was 38.5 months and median PFS 28,6 months, with only three cases of grade 3 toxicity. However, this study did not focus specifically on central lesions and the utilized technique was 3D‐CRT.^33^ More informative were studies which analyzed ultracentral tumors treated with 12–15 fraction schedules. A schedule of 60 Gy in 12 fractions (4 fractions per week) had high local control—1‐ and 2‐year LC rates were 98% and 85%, respectively. 1‐and 2‐years OS rates were 77% and 52%, respectively. However, grade 3+ toxicity was observed in 21% patients, of which 14% died of bronchopulmonary hemorrhage. Dmean BED3 higher than 91 Gy in the main bronchus significantly increased the risk of grade 3+ toxicity.^34^ Another study showed that this fractionation was connected with median OS of 15.9 months and 1‐, 2‐, and 3‐year OS rates were 61.5%, 28.7%, and 20.1%, respectively. Grade 3+ toxicity was experienced in 38% patients and 21% of patients died possibly in relation to RT.^35^ Patients treated with 5–15 fractions up to 45–60 Gy delivered every other day had 1‐, 2‐ and 3‐year OS rates of 78.6%, 64.5% and 53.1%, respectively. Grade 3+ toxicity was present in 21.6% patients and 11% patients experienced likely or possible RT‐related death (radiation pneumonitis, hemorrhage). Fractionation schemes were not associated with grade 3+ toxicity‐free survival.^36^

The International Stereotactic Radiosurgery Society (ISRS) summarized evidence of SBRT in ultracentral tumors in a systematic review and meta‐analysis. In the guidelines the authors recommend 60 Gy in eight fractions with highest priority given to organs at risk and avoiding hot spots in critical structures. Special attention should be given to patients using anti‐platelet/anticoagulation drugs and with endobronchial tumors resulting in non‐SBRT schedules, for example, 60 Gy in 15 fractions. Most studies analyzed treated patients on consecutive days. Concomitant SBRT with immunotherapy requires at least 2 days interval and VEGF inhibitors 2–3 weeks. Patients with interstitial lung disease (ILD) have higher risk of pneumonitis—treatment preparation should limit the volume of normal lung—V20 < 10%.^13^

### Summary of evidence for SBRT in central and ultracentral tumors

Recent studies have focused on various hypofractionation schedules. The majority are based on 5 × 8.5 Gy–12 Gy schemes. High toxicity remains the main obstacle for SBRT in this localization, mainly due to damage of normal cells in the bronchi or lungs that could not be repaired after the ablative dose. Another problem is associated with higher clinical manifestation of inflammation than in peripheral localization. The definition of an ultracental location varies in multiple studies. SBRT of tumors largely invading the proximal bronchial tree and volume abutting a large vessel should not be analyzed together as a large difference in terms of expected toxicity in those locations is expected. Therefore in clinical practice special care and different dose fractionation should be selected for tumors abutting, invading or volume abutting the proximal bronchial tree as grade 5 toxicity comes mainly from this organ at risk (OAR). Central tumors should be treated with 4–5 fractions daily whereas ultracentral with 8–15 fractions daily.

The SUNSET trial may provide the answers about the maximum tolerated dose (MTD) in patients with ultracentral T1‐3 (<6 cm) N0 M0 NSCLC.^37^ [Correction added on 26 September 2023, after first online publication: Table 1 citation has been deleted in the preceding sentence and a new sentence to cite Table 1 ‘Table 1 summarizes analyzed studies.’ has been added.] Table 1 summarizes analyzed studies.

**TABLE 1:** Analyzed studies.

Author	Number of patients	Dose‐fractionation (Gy/fractions)	BED (Gy)	Prescripton method	Technique	Treatment pattern	Toxicity	Fatal toxicity	Local control (%)
Peripheral tumors									
Wood et al^17^	412	50–60/3–10	75–151.2	conformal plans: dose prescribed to the 80% isoline VMAT: 95% of PTV received 100% PD	4D‐CT/non‐4D‐CT	Consecutive days/alternate days	1% rate of G3‐4 toxicity	0	NA
Jain et al^18^	54	48–52/4	NA	99% of ITV received PD, 99% PTV received 95% of PD	4D‐CT	4 consecutive days/every 3rd or 4th day	Toxicity G2 + 55.6% (4‐days group) 33.3% (11‐days group)	NA	NA
Jun et al.^19^	155	30–60/1–3	NA	99% of PTV received greater than or equal to 90% PD	4D‐CT	nr	No G3 + in the first 6 months	0	1‐year LC‐95.1% for the SF‐SBRT 92.7% for the TF‐SBRT
Singh et al^20^	98	30–60/1–3	NA	According to RTOG 0915 and RTOG 0236	4D‐CT/non‐4D‐CT	3 fractions – at least 40 h interval	16% (1 fraction), 12% (3 fractions) G3 toxicity no G4‐5	0	2‐year LC 94.9% (1 fraction) 97.1% (3 fractions)
Videtic et al^21^, ^22^	94	34–48/1–4	NA	95% of PTV ≥90% of PD	4D‐CT/non‐4D‐CT	Once daily	10.3% (1 fraction), 13.3% (4 fractions) G3 + in long‐term analysis: 2.6% (1 fraction) 11.1% (4 fractions) G3+	2 cases, no new G5 toxicity in longer follow up	1‐year LC 92.7% (1 fraction) 97% (4 fractions)
Videtic et al^23^	229	30–34/1	NA	According to RTOG 0915 and RPCI‐124407	4D‐CT/non‐4D‐CT	Once daily	Any grade 27.1%, no G4‐5	0	NA
Central/ultracentral tumors									
Roach et al^29^	74	11/5	NA	95% PTV covered by PD and at least 99% PTV received 90% of PD	4D‐CT	NA	Acute 6% G3‐4 late 27% G3 12% G4	4%	2‐year LC 85%
Lindberg et al^30^	65	56/8	Mean BED 95.2	Dose prescirbed to 67% isodose	4D‐CT/other	Max 4/week	34% G3+	15%	2‐year LC 83%
Cho et al^31^	131	60/15–20	78–84	At least 97% of PD to 95% of the CTV	4D‐CT/other	Daily	64.5% pneumonitis G1‐5 37.1% esophagitis G1‐2	0.8%	2‐year LC 62.6%
Chang et al^32^	100	50–70/4–10	112.5/119	Prescribed to a dose of 50 Gy to PTV, 85%–95% isodose line	4D‐CT	Daily	Chest‐wall pain (18% G1 13% G2) pneumonitis (11% G2 1% G3)	0	3‐year LC 96.5%
Lodewegs et al^34^	72	60/12	137	95% of PTV received PD (PTV *D*_95%_ ≥ 60 Gy) 99% of PTV received at least 90% PD (PTV *D*_99%_ ≥ 54 Gy)	3D‐CT/4D‐CT	4/week	21% G3 +	14%	1‐/2‐year LC 98% and 85%
Bogart et al^33^	39	70/17–29	NA	Dose prescribed to isocenter 95% isodose line required to encompass the PTV	3D‐CRT/non‐4D‐CT	Once daily	Only G3	0	NA
Tekatli et al^35^	47	60/12	90	95% and 99% of the PTV, received 100% and 90% of PD	4D‐CT	4/week	38% G3+	21%	NA
Wang et al.^36^	88	45–60/5–15	≥84 Gy	NA	4D‐CT	NA	22% G3+	11.4%	NA
Finazzi et al.^38^	50	5–18/3–12	≥100Gy	95% PTV covered by PD (PTV V100% ≥95%)	MR‐guided SBRT	NA	G2 + 30% G3 + 8%	0	1‐year LC 95.6%

Abbreviations: 3D‐CRT, three‐dimensional conformal radiotherapy; 3D‐CT, three‐dimensional computed tomography; 4D‐CT, four‐dimensional computed tomography; BED, biologically effective dose; LC, local control; NA, no available; PD, prescription dose; PTV, planning target volume; SF‐SBRT, single fraction stereotactic body radiation therapy; TF‐SBRT, three fractions stereotactic body radiotherapy.

### New approach to SBRT in central location

Stereotactic MR‐guided radiation therapy (SMART) is a novel approach that reduces high grade toxicity with an acceptable level of local control. SABR with real‐time MR guidance and on‐table plan adaptation delivers highly precise treatment in challenging patients. In a recent study, SMART was used in patients with central lung tumors, significant tumor motion, prior radiotherapy and lung resection, with multiple synchronous lung tumors and interstitial lung disease. The authors used four risk‐adapted dose‐fractionation schedules: 8 × 7.5 Gy, 5 × 11 Gy (two of the most common schedules), 3 × 18 Gy and 12 × 5 Gy.

Treatment planning required a 17‐s breath‐hold 3D MR scan. A breath‐hold planning CT scan was used for dose calculation purposes and to verify tumor properties. Before daily treatments, a new breath‐hold 3D MR scan was made. If needed, GTV and OARs contours generated on pretreatment MRI scans were corrected based on new MRI. PTV was created by adding isotropic 5 mm margin to the breath‐hold GTV. A clinician approved “predicted plan”—plan recalculated on the anatomy of the day. SABR was delivered with continuous tracking of the GTV on MR. To improve gating efficiency visual feedback was provided on an in‐room monitor, which projected the GTV and a gating window in real‐time. Interfractional changes in clinician contoured GTV were minimal and in 41% of fractions GTV was not corrected. In 75% of fractions changes in PTV were <1 cm^3^. Reoptimized plans were chosen in 91% fractions, mostly due to improved PTV coverage, OARs sparing or both. Daily on‐table plan adaptation allowed 95% of the PTV of the BED >100 Gy in the whole treatment in 92.6% tumors to be delivered. The average gain in PTV coverage by the prescription dose (V100%) was 4.4% per fraction. Mean PTV volume was 9 cm^3^. One patient did not complete the planned SMART treatment. Median follow‐up was 21.7 months. LC, OS, and disease‐free survival (DFS) rates at 12 months were 95.6%, 88.0%, and 63.6%, respectively. Rates of grade 2–3 toxicity were 30% and 8%, respectively, including chest wall pain and pneumonitis. No grade 4 or 5 toxicities were observed. This technique requires more time than standard SBRT. Median treatment time on an MR Linac was 49 min.^38^ Currently, the LUNG STAAR trial is evaluating the role of SMART in ultracentral tumors.

### SBRT after prior irradiation

Another obstacle is SBRT after prior radiation. ASTRO evidence‐based guideline recommends salvage SBRT in selected patients after conventionally fractionated radiotherapy as well as SBRT. SBRT offers good LC and survival but patients must be informed about potential toxicities (included fatal complications).^27^

An international Expert Survey on the Indication and Practice of Radical Thoracic Reirradiation for NSCLC suggests reirradiation should be divided into two groups:^1^ reirradiation for local relapse—salvage reirradiation—with high degree of OAR (organs at risk) dose overlap and^2^ new primary NSCLC with little or no overlap between two courses of radiation but increased volume of irradiated lung. Reirradiation should be used cautiously in patients who experienced grade 3+ toxicity in previous radiotherapy. Retreatment should be avoided in patients with interstitial lung disease. Experts could not agree on the minimal interval between radiotherapy courses. However, the majority of them would reirradiate after 6 months. Observation, systemic treatment and surgical resection should be considered if the interval is shorter. SBRT is preffered in small, non‐ultracentral lesions with minimal overlap of the previously treated OARs. Meeting OAR dose constraints remains the highest priority. Adding a margin to GTV to create CTV did not reach consensus. In reirradiation cases additional margin requires special consideration due to possible toxicity and could be chosen for selected cases. Maximum expansion of GTV to CTV can be 5 mm. If OAR dose constraints cannot be met, the CTV margin could be reduced or omitted. There is a lack of evidence for dose constraints for lung reirradiation. No consensus was reached on the constraint for the proximal bronchial tree. Suggested values for a desired Dmax EQD2 of <80 Gy with an absolute maximum dose of 105 Gy (α/β = 3) reached 66.7% agreement.^39^

#### Dose‐fractionation of re‐SBRT

A review of lung reirradiation studies revealed that the most common schedule of re‐SBRT ranged between 17 and 85 Gy in 1–10 fractions. Any dose and fractionation that can safely deliver a BED >100 Gy to the tumor is acceptable for radical reirradiation with SABR. In cases where OAR dose constraints for SBRT cannot be met, treatment with conventionally fractionated radiotherapy might be used. Doses include 60 Gy in 30 fractions and 55 Gy in 20 fractions once daily.

#### Outcomes of re‐SBRT

SBRT reirradiation has previously been found to be associated with median OS 12–40 months, 1‐, 2‐year OS of 48.9%–84.6% and 33%–74.4%, respectively. In this study, 1‐ and 2‐year LC were 67% and 54%, respectively. Overall, 2‐year PFS ranged from 26% to 37%. Conventionally fractionated reirradiation was associated with the median OS of 7,1–13,5 months, 2 years OS rates of 23%–51%. Median locoregional PFS was 6.5 months, with 1‐ and 2‐year locoregional PFS of 51% and 42%. Worse outcomes in patients treated with conventionally fractionated radiotherapy compared to SABR could be explained by lower BED or higher volume of the disease. Toxicity depends on radiotherapy technique, irradiated volume, dose fractionation, patient comorbidities and disease location. The degree of isodose overlap is a contributing factor in the toxicity. The most common toxicity is pneumonitis (7%–25% patients).^40^

In another study, re‐SBRT of 5 × 9–10 Gy up to 45–50 Gy (3 fractions per week) had a good safety profile (no G4‐5 toxicity) and LC of 92% and 1‐ and 2‐year OS rate of 80% and 36%, respectively.^41^

It has also been reported that schedules of 30–60 Gy in 3–5 fractions (median BED 132 Gy) delivered over a median 9 days had OS at 2‐years 68.6% and no toxicities G4‐5.^42^

In conclusion, studies suggest that in peripheral tumors shorter interval between fractions does not increase toxicity. According to ESTRO guidelines, as well as prospective and retrospective studies, treatment on consecutive days is recommended. In peripheral and ultracentral tumors recommendations define specific dose‐fractionation depending on tumor size and localization. In peripheral tumors up to 5 cm radiosurgery is recommended. In tumors abutting the chest wall—5 × 11 Gy, in central tumors >1 cm from main bronchi—5 × 10 Gy and in ultracentral tumors—8 × 7.5 Gy. In gross endobronchial infiltration the schedule of 8 × 7 Gy should be avoided due to increased risk of toxicity (HILUS). Especially in coexistence of risk factors for inducing hemoptysis such patients should be treated with extreme caution. According to the ASTRO recommendation in ultracentral tumors short schedules of <3 fractions should be avoided. Re‐SBRT is often used but it should be avoided in ILD and only >6 months after prior treatment.

## AUTHOR CONTRIBUTION

Maciej Harat made a substantial contribution to the concept and design of the article. Izabela Zarębska was responsible for collecting and interpreting the data as well as drafitng the manuscript. Both authors analyzed the evidence and revised critically the content. The final version of the manuscirpt was accepted by both authors.

## CONFLICT OF INTEREST STATEMENT

The authors declare that they have no competing interests.
